# Circular Economy trifft urban-regionale Resilienz – Synergien für eine nachhaltig-anpassungsfähige Stadtentwicklung

**DOI:** 10.1007/s00548-022-00815-0

**Published:** 2022-12-02

**Authors:** Martina Fromhold-Eisebith

**Affiliations:** Lehrstuhl für Wirtschaftsgeographie, Geographisches Institut, RWTH Aachen, Templergraben 55, 52056 Aachen, Deutschland

**Keywords:** Urbane Resilienz, Regionale Resilienz, Kreislaufwirtschaft, Innovation, Indikatorik, Urban resilience, Regional resilience, Circular economy, Innovation, Indicators

## Abstract

Um der Circular Economy bessere Möglichkeiten zur Integration in stadtregionale Strategien zu verschaffen, bietet sich die Verknüpfung mit weiter gefassten Zielen der Resilienz an. Die Konzepte der Urbanen und Regionalen Resilienz werden auch in Deutschland zunehmend als Ansatz für Zukunftsfähigkeit diskutiert. Sie sollen die Robustheit, Anpassungs- und Erholungsfähigkeit von Städten bzw. Regionen im Angesicht vielfältiger Krisen stärken, verknüpft mit Zielen der nachhaltigen Entwicklung. Der Beitrag zeigt einerseits auf, in welcher Hinsicht die kombinierte Sicht auf urban-regionale Resilienz einen geeigneten Rahmen für die effektive Einbettung systemischer Ansätze der Circular Economy ins stadträumliche Gefüge schafft. Andererseits wird erläutert, wie im Gegenzug auch die resiliente Abfederung umwelt- und wirtschaftsbezogener Risiken besser gelingen kann, wenn kreislaufwirtschaftliche Elemente integriert werden. Nach konzeptionellen Überlegungen zu synergetischen Wechselwirkungen dieser Prozessfelder dient das Beispiel der Stadt Aachen zur Illustration der zweckmäßigen Kombination von „Circular City“ und resilienzbezogenen Zielen. Dies macht deutlich, wie bedeutsam ein Fokus auf Innovationen für solche Strategieansätze ist. Am Ende werden Empfehlungen zur Gestaltung einer resilient-zirkulären Stadtökonomie formuliert.

## Abstract (en)

To provide the Circular Economy with better opportunities for integration into city-regional strategies, linkages with broader objectives of resilience are solicited. The concepts of Urban and Regional Resilience are also increasingly discussed in Germany, as an approach to future viability. They are expected to bolster the robustness, adaptability, and recovery capabilities of cities and regions in the face of multiple crises, connected to objectives of sustainable development. This article, on the one hand, shows in which respects a combined view of urban-regional resilience establishes a suitable framework for effectively embedding systemic approaches of the Circular Economy into city-regional contexts. On the other hand, it explains how, in turn, the resilient cushioning against environment- and economy-related risks can be more successful if elements of the Circular Economy are integrated. After conceptual considerations of the synergistic interdependencies of these process fields, the example of the City of Aachen serves to illustrate the appropriate combination of ‘Circular City’ and resilience-related objectives. It elucidates how important a focus on innovation is for such strategic approaches. Finally, recommendations are made for designing a resilient-circular urban economy.

## Ausgangslage und Problemstellung

Um den wachsenden Herausforderungen einer nachhaltigen Entwicklung von Städten bzw. Regionen zu begegnen, sollte zur Stützung der Circular Economy der Resilienz-Ansatz stärker beachtet werden. Auch er adressiert wichtige Nachhaltigkeitserfordernisse, konkret die Anpassung räumlicher Systeme an Schocks und Krisen (UNDP 2021). Resilienz gewinnt als Leitidee für regionale Zukunftsfähigkeit in Deutschland an Bedeutung (BMI 2021), wurde bislang aber kaum mit Zielen der Circular Economy verbunden (Fratini et al. 2019; EMF 2022). Letztere ist dabei – über die schmalere Bedeutung des deutschen Terminus Kreislaufwirtschaft hinausgehend – explizit als Bündel diverser „R-Strategien“ zu verstehen (Potting et al. 2017 nennen zehn, wie u. a. Refuse, Reduce, Repurpose, Recycle, Recover), die verschiedene wirtschaftlich-gesellschaftliche Praktiken betreffen (Korhonen et al. 2018). Die Leitfrage dieses Beitrags ist somit: Wie können Strategien städtisch-regionaler Resilienz konstruktiv Elemente der Circular Economy einbinden, sodass beide Aktivitätsfelder bei der Stützung einer ökologisch, ökonomisch und sozial nachhaltigen Stadtentwicklung synergetisch zusammenwirken?

Hierfür werden Verknüpfungsmöglichkeiten von Urbaner/Regionaler Resilienz und Circular Economy aufgezeigt, im Sinne des Slogans „Circular cities: thriving, liveable, resilient“ (EMF 2022). Einerseits wird erklärt, inwiefern Aspekte der Resilienz einen guten Rahmen für die Einbindung der Circular Economy bieten, und andererseits erläutert, wie Letztere zur resilienten Abfederung akuter Krisen beitragen kann. Zentrale Annahmen werden in einem Synergie-Modell zusammengeführt und mit Überlegungen zur Indikatorik verbunden. Illustration bietet das Beispiel der resilienten „Circular City“ Aachen. Der Beitrag schließt mit Handlungsempfehlungen für ein konstruktives Zusammenspiel öffentlicher Akteure (v. a. Stadtverwaltung) mit Privatwirtschaft und Bürgerschaft.

## Resilienz-Ansätze als förderlicher Rahmen für Circular Economy

Indem Resilienz die Fähigkeit dynamischer Systeme beschreibt, flexibel auf Krisen zu reagieren und sich anzupassen, sind Bezüge zu natürlichen Ökosystemen bis hin zu Sets ökonomischer und sozialer Akteure denkbar. Dies baut Brücken zu mehreren planungs- und nachhaltigkeitsrelevanten Teilsystemen, wie der Circular Economy. Weil jene als regionale Aufgabe zu begreifen ist (Fromhold-Eisebith 2022), mit Fokus auf städtische Räume bzw. Circular Cities (OECD 2020; Circle Economy et al. 2021), liegt die Einbettung in Ansätze urban-regionaler Resilienz nahe.

Vor allem die kombinierte Sicht auf raumbezogene Resilienz-Varianten schafft gute Ansatzpunkte für die Circular Economy. Urbane Resilienz stellt Anpassungsbedarfe von städtischen (Infra‑)Strukturen, Bevölkerung und Governance angesichts von Natur- bzw. Klimarisiken ins Zentrum (Brantz und Sharma 2020; UNDP 2021; für eine – hier ausgeklammerte – kritische Debatte siehe Meerow und Newell 2019). Regionale Resilienz hingegen betont ökonomische Systemdynamiken unter dem Einfluss wirtschaftsrelevanter Schocks (Bristow und Healy 2020; Martin und Sunley 2015). Zwar gilt auch bei Urbaner Resilienz die Ökonomie als ein Baustein unter vielen, doch bietet erst die Synthese beider Konzepte gute Optionen zur zweckmäßigen Integration der Circular Economy ins stadträumliche Gefüge.

Inwiefern weist Resilienz generell kreislaufwirtschaftliche Bezüge auf? Wesentlich ist, dass Akteursgefüge und Institutionen einer resilienten Stadtregion so zu gestalten sind, dass sie besonders robust und anpassungsfähig auf Krisen reagieren können (Bristow und Healy 2020; UNDP 2021). Weil auch die Abhängigkeit von exogenen Ressourcen eine regionale Verwundbarkeit begründet (wie aktuell die gefährdete Energieversorgung Deutschlands zeigt), tragen regionale Stoffkreisläufe zur Resilienz bei, indem sie Abhängigkeiten mildern. Ein weiteres Argument betrifft die regionale Reaktionsfähigkeit auf Einschränkungen bei Ressourcenverbrauch und Konsum, wie sie im Zuge von Nachhaltigkeitspolitiken künftig wohl zunehmend verordnet werden. Gut eingespielte Ansätze der Circular Economy sorgen dafür, dass auch solche Resilienz-Herausforderungen besser gemeistert werden.

Das Konzept Urbaner Resilienz bietet strategierelevante Ansatzpunkte, indem es Fähigkeiten städtischer Akteure und Systeme betont, verschiedene Umweltrisiken abzufedern (Ribeiro und Gonçalves 2019; Wang et al. 2018). Dies betrifft Ver‑/Entsorgungsbereiche der Stadt sowie Bau- und Infrastrukturelemente, die robuster, anpassungs- und erholungsfähiger werden sollen. Dabei geht es eher um baulich-physische, natürliche, institutionelle und soziale Systembereiche als die Wirtschaft. Doch sind auch ökonomische Zusammenhänge für die Krisenanpassung wichtig: Die städtische Gewerbeentwicklung stützt maßgeblich Daseinsgrundfunktionen wie Arbeiten, Wohnen, Bildung, Versorgung und Mobilität. Über die regionalen Steuereinkünfte ermöglicht sie die Finanzierung öffentlicher Aufgaben in vielen Nachhaltigkeitsfeldern.

Das wirtschaftsgeographische Konzept der Regionalen Resilienz bietet deshalb wichtige komplementäre Perspektiven: Es adressiert die Fähigkeit einer Regionalwirtschaft, gegenüber markt-, wettbewerbs- oder nachhaltigkeitsbezogenen Schocks robust zu bleiben bzw. sich rasch zu erholen, indem sie bei Branchenstruktur, sozialen und institutionellen Konstellationen anpassungsfähig ist und – gestützt auf die produktive Nutzung materieller, humaner und umweltbezogener Ressourcen – den Pfad zu einer nachhaltigeren Entwicklung einschlagen kann (nach Martin und Sunley 2015, S. 13). Als wesentliche Treiber gelten neben flexiblen Akteurssettings auch unternehmerisches Engagement und Kreativität, variable Vernetzungen und interinstitutionelle Synergien (Bristow und Healy 2020). Bedeutsam ist vor allem die längerfristige Anpassungsfähigkeit an immer neue Herausforderungen. Relevant für Resilienz sind Branchenstruktur, Dynamik und Wettbewerbsfähigkeit, Exportstärke der Regionalwirtschaft, Produktivität und Effizienz, Humankapital, Institutionenbesatz, Innovations- und Technologiepotenziale, Politikregime sowie Geschäftsklima und unternehmerische Kultur (Martin und Sunley 2015).

Um Elemente der Circular Economy in breiter nachhaltigkeitsbezogene stadtregionale Anpassungsstrategien zu integrieren, ist die Zusammenführung beider Resilienz-Varianten zielführend. Einerseits schafft der auf Infrastruktur sowie öffentliche Ver‑/Entsorgung gerichtete Blick der Urbanen Resilienz gute Möglichkeiten, Bereiche wie Abfall- und Abwasserbehandlung, Energieeffizienz und geteilte Mobilität als wichtige Facetten der Circular Economy ins Stadtgefüge einzubetten. Dies weist adäquat den für öffentliche Aufgaben zuständigen Akteuren und Institutionen die Mitverantwortung für Kreislaufaspekte im Zuge einer urbanen Resilienz-Strategie zu. Andererseits werden die auf ökonomische Prozesse und zwischenbetriebliche Wertschöpfungsketten gerichteten Aspekte der Circular Economy durch Bezug auf Regionale Resilienz gut gerahmt und unterstützt, mit Betonung unternehmerische Akteure und ihres Zusammenwirkens. Hier spielen außerdem Ansätze der auf Materialsubstitution, Effizienz und Recycling gerichteten Innovation bzw. Technologieentwicklung mit hinein, bei konstruktiver Einbindung örtlicher Forschungseinrichtungen und Hochschulen zum Nutzen der resilienten Kreislaufwirtschaft. Insgesamt lassen sich mit Zielen der urban-regionalen Resilienz also diverse Prozessfelder der Circular Economy verbinden.

## Circular Economy als Förderfaktor für urban-regionale Resilienz

Im Gegenzug fördert die Circular Economy auch die örtliche Resilienz im Zuge beidseitiger Synergiepotenziale. Sie hilft verschiedene Arten von Krisen abzufedern, die akut die Reaktionsfähigkeit von Städten und ihren Ökonomien herausfordern:

- Globale Pandemien werden auch nach COVID-19 immer wieder Wirtschaftskrisen auslösen. Bei weiträumigen Lieferketten kann dies in deutschen Regionen zu Versorgungsengpässen für Importbedarfe führen. Die Circular Economy bietet Möglichkeiten, durch verringerten Ressourcenbedarf und stärker regionalisierte Wertschöpfungssysteme die externen Abhängigkeiten resilient zu verringern.
- Regionen in reifen Industriestaaten sind fortlaufend mit Wettbewerbskrisen konfrontiert, weil Produktions- wie Dienstleistungsbetriebe gegen die Konkurrenz vor allem aus China nicht mehr bestehen können. Zwar kann auch die Circular Economy kaum die typisch stadtregionalen Kostennachteile bei Flächen, Gehältern und Wohnraum mildern. Doch könnte das durch zirkuläre Praktiken und Sharing Economy wachsende Engagement für mehr Konsum regionaler Produkte trotz höherer Preise zur Abfederung beitragen.
- Der Klimawandel birgt wachsende Risiken für städtische Infrastrukturen und Aktivitäten, wie für Urbane Resilienz zentral. Auch die Stadtökonomie ist durch Naturkatastrophen gefährdet, welche die in Tallagen konzentrierten Gewerbe- bzw. Einzelhandelsstandorte und ihre Ver‑/Entsorgungsbereiche zerstören. Die oft dezentralen, breiter im Stadtraum verteilten Bausteine einer Circular Economy (z. B. Energieversorgung) könnten sowohl die örtliche Robustheit gegenüber solchen Ereignissen stützen als auch für eine rasche Erholung und den Ersatz beeinträchtigter Funktionen durch Alternativen sorgen.
- Im Klimawandel steht außerdem die rasche Dekarbonisierung der Regionalwirtschaft und Energieversorgung an. Dabei fordern vor allem verschärfte gesetzliche Ansprüche Resilienz heraus, indem Ziele der CO_2_-armen, ressourceneffizienten Wirtschaftsweise recht plötzlich Änderungen verlangt (z. B. neue Regelungen zu Emissionseindämmung, CO_2_-Bepreisung und Nutzung regenerativer Energien). Außerdem können disruptiv wirkende Innovationssprünge im Bereich nachhaltiger Produktionsweisen ganze lokalisierte Produktionssysteme betreffen. Hier bietet die Circular Economy bezogen auf alle R‑Strategien (Potting et al. 2017) die entscheidende Basis, um die stadtregionale Ökonomie resilienzfördernd umzubauen.

**Abb. 1:**
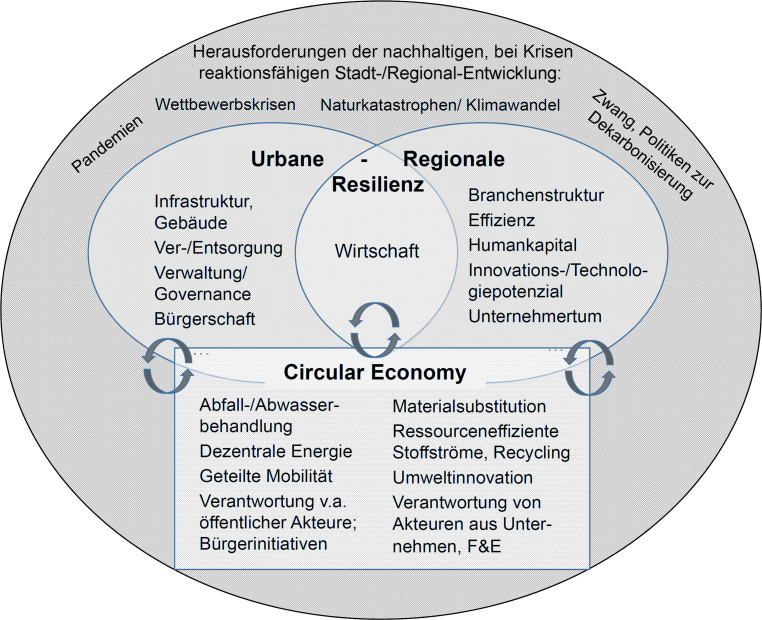
Modell des synergetischen Zusammenspiels von Urban-Regionaler Resilienz und Circular Economy. (Eigene Darstellung)

Die Überlegungen lassen sich in einem Modell zusammenführen, das Synergien urban-regionaler Resilienz mit Bereichen der Circular Economy aufzeigt (vgl. Abb. 1). Ein solches Modell sollte helfen, in der Praxis die Verschränkung dieser Aufgabenfelder voranzutreiben und zumindest mehr Bewusstsein für entsprechende Strategieoptionen zu wecken.

Zur effektiven Einbindung der Circular Economy in eine resiliente Stadt‑/Regionalentwicklung ist auch die indikatorbasierte Erfassung anzureichern, die wichtige Zielmarken und Bewertungsmaßstäbe setzt. Die ohnehin systemischen Messansätze zur Resilienz bieten bereits Ansatzpunkte. So integriert die Erfassung von City Resilience (Da Silva 2013) Dimensionen zu Umwelt, Infrastruktur und Governance, die sich für ergänzende Messgrößen anbieten, etwa zu Recyclingquoten bzw. Abfallverwertungsarten, Selbstversorgungsgrad bei Energie, Gebäudedämmung, Investitionen in umweltschonende Mobilität oder Anzahl lokal agierender Sharing-Initiativen (Circle Economy et al. 2021). Auch die differenzierte Indikatorik zur Regionalen Resilienz berücksichtigt schon Nachhaltigkeitsaspekte (z. B. Resilienz-Barometer Deutschland; agiplan 2014), dazu Innovation und Bildung. Mehr Fokus auf Circular Economy schaffen Daten etwa zu regionalen F&E-Ausgaben für Kreislauftechnologien, zu Investitionen in die Herstellung kreislauffreundlicher Produkte, zum zwischenbetrieblichen Stoffstromaustausch oder Aufkommen „grüner“ Start-ups.

## Beispiel Aachen: Ansatzpunkte für die resiliente Circular City

Wie Synergien von Resilienz und Circular Economy in Gang kommen könnten, illustriert das Beispiel der Stadt Aachen. Schon länger hat hier der Niedergang traditioneller Branchen dazu gezwungen, im Sinne regionaler Resilienz neue wirtschaftliche Wege einzuschlagen. Dabei haben sich die Aktivitäten in hohem Maße auf Innovationsimpulse aus den regionalen Hochschulen und Forschungseinrichtungen gestützt (Fromhold-Eisebith 2012). Die regionalökonomische Anpassungsfähigkeit wird so schon seit Jahrzehnten vor allem über die Förderung technologieorientierter Unternehmensgründungen weiterentwickelt.

**Abb. 2:**
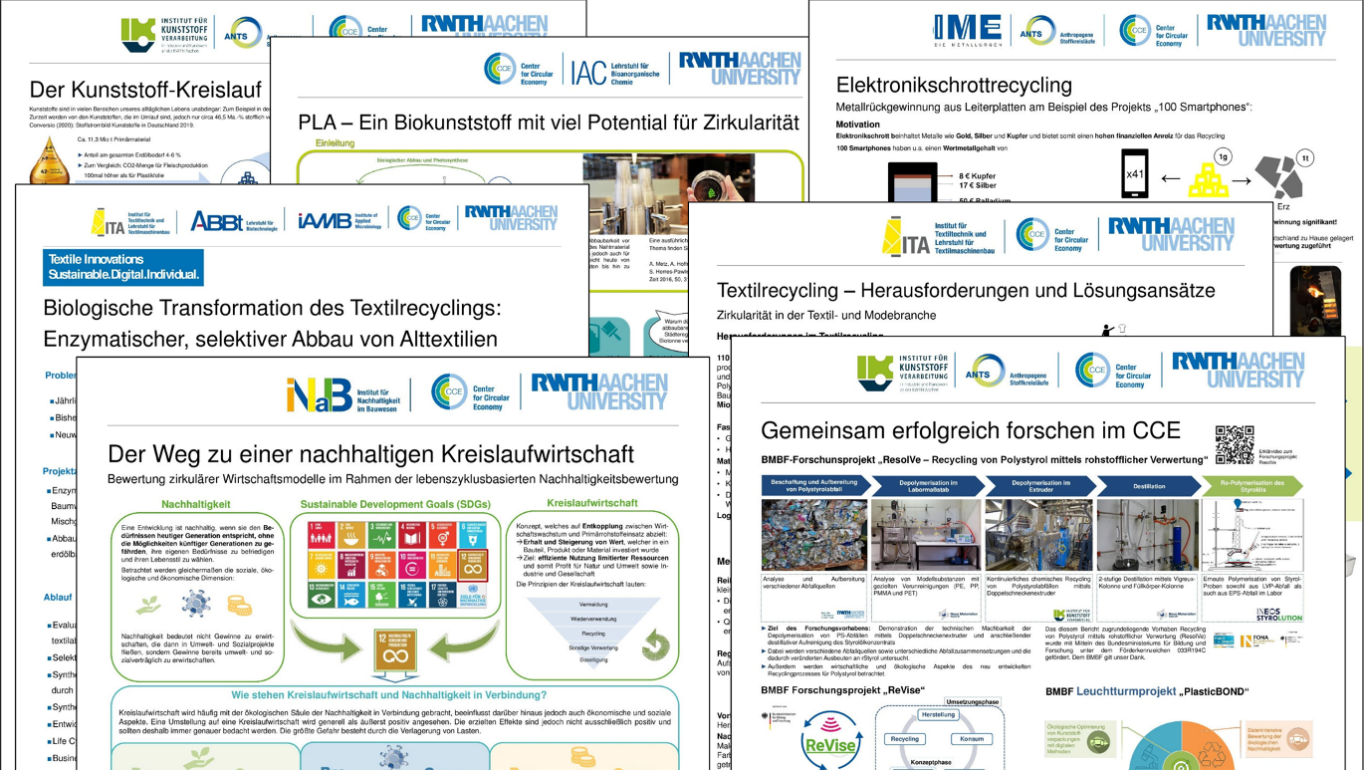
Forschungsprojekte im Rahmen des Centers for Circular Economy der RWTH Aachen. (Fotocollage: M. Fromhold-Eisebith)

Regionale Kompetenzen sollen jetzt dezidiert auf Circular Economy ausgerichtet werden, verankert im Beitritt der Stadt Aachen zur „Circular City Declaration“ im Oktober 2021. Wesentlicher Baustein ist auch hierbei das Innovationspotenzial der RWTH Aachen, wo ein neu etabliertes Center for Circular Economy (CCE) verschiedene F&E-Vorhaben zu Materialeinsparung und Kreislaufnutzung integriert (vgl. Abb. 2).

**Abb. 3:**
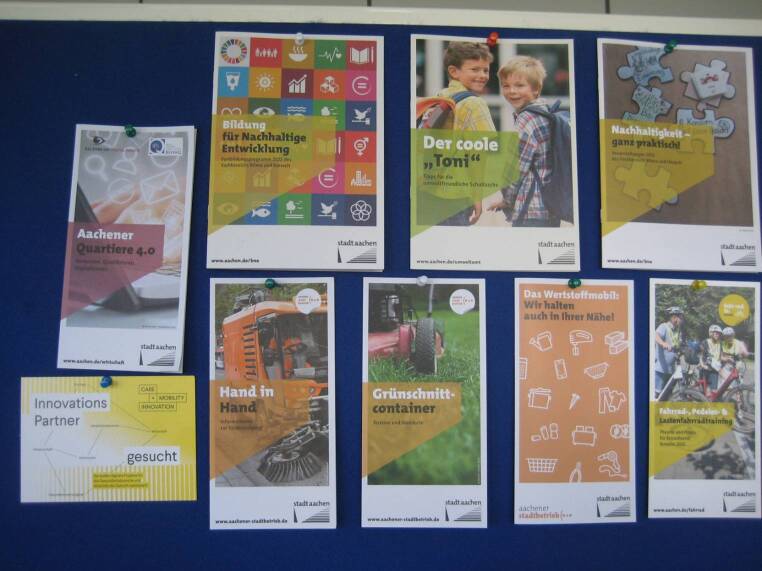
Patchwork von Ansätzen der Stadt Aachen zu Bereichen der Circular Economy. (Foto: M. Fromhold-Eisebith)

**Abb. 4:**
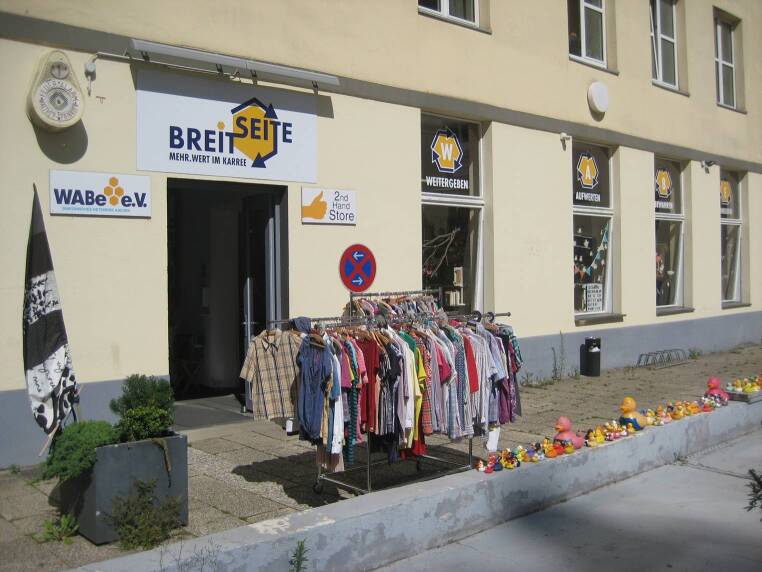
Eine der vielen bürgerschaftlichen Secondhand-Initiativen in Aachen. (Foto: M. Fromhold-Eisebith)

Unter Federführung der Stadt Aachen soll außerdem die sparsame Energie- und Ressourcennutzung unterstützt werden. Im Bündnis mit Aachener Hochschulen, Forschungsinstituten, der IHK und regionalen Energieversorgern will man die „Wärmewende“ vorantreiben, bis zur Klimaneutralität in 2030. Der „Maßnahmenplan 2025“ sieht vor, bis 2024 in den Photovoltaikausbau kommunaler wie sonstiger Gebäude sowie in die energetische Gebäudesanierung rund 80 Mio. € zu investieren (Der neue Kämmerer 2021). Um die resiliente Circular City Aachen breiter aufzustellen, wäre allerdings auch die Integration sonstiger Aktivitäten wichtig. Noch wirken die kreislaufbezogenen Vorhaben der Stadt wie Patchwork, ohne übergreifende Strategie (Abb. 3). Sie müssten auch mehr bürgerschaftliches Engagement einbinden, wie die zahlreichen Secondhand-Shops in der Stadt (vgl. Abb. 4 für ein Beispiel). Interessant ist speziell die Initiative Regionale Resilienz Aachen e. V., deren jetzt anlaufende Kampagne zirkuläre Ansätze explizit betont (Regionale Resilienz Aachen e. V. 2022).

## Fazit: Empfehlungen zur Gestaltung einer resilient-zirkulären Stadtökonomie

Abschließend werden zur propagierten Einbettung der Circular Economy in Strategien der urban-regionalen Resilienz Handlungsempfehlungen formuliert. Dabei müssen die Akteursgruppen Stadtverwaltung, Wissenschaft und Bildung, dazu Betriebe, die innovative Ideen aufgreifen und (experimentell) umsetzen, außerdem bürgerschaftliche Initiativen sowie Allianzen privater und öffentlicher Akteure eng zusammenwirken. Die Circular Economy ist konsequent mitzudenken, wenn es um den krisenrobusten Infrastrukturausbau und die zukunftsfähige Wirtschaftsentwicklung geht. Die folgenden Strategiefelder sind für die Gestaltung einer resilient-zirkulären Stadtökonomie besonders wichtig:

- Das auf Resilienz bezogene Monitoring zu Krisenrisiken und potenziellen regionalen Gefährdungen sollte mit einem systematischen Monitoring auch der Circular-Economy-Optionen verbunden werden.
- Bei der Bildung regionalwirtschaftlich relevanter Institutionen (z. B. Förderprogramme, Forschungsverbünde) ist das Erfordernis der Flexibilität und Anpassungsfähigkeit immer mit der Ausweitung kreislaufwirtschaftlicher Praktiken zu verschränken.
- Alle für urban-regionale Resilienz unterstützten Akteursvernetzungen sind dezidiert daraufhin zu überprüfen, inwiefern damit auch die vielen R‑Strategien (Potting et al. 2017) der Circular Economy vorangebracht werden können.
- Die regionale Innovations- und Technologieförderung ist für Resilienz wie Circular Economy hoch bedeutsam, um vor Ort maßgeschneiderte Lösungen bereitzustellen. Deshalb sollten Anreize und Freiräume für zukunftsweisende Forschungs‑/Bildungsaktivitäten geschaffen werden (z. B. *white spaces* F&E-Kooperationen, die ohne thematische Bindung explorativ neue Wege ausloten können).
- Möglichkeiten der Digitalisierung städtischer bzw. stadtökonomischer Funktionen sollten konsequent genutzt bzw. vorangetrieben werden, mit Einsatz IT-basierter Koordinations- und Steuerungsmöglichkeiten für alle städtischen Ver- und Entsorgungsbereiche sowie diverse Stoffströme des urbanen Metabolismus.
- Bei der regionalen Branchen- bzw. Clusterförderung sollten breitere, von früheren Abhängigkeiten wegführende Aktivitäten aufgegriffen werden, wofür die Bereiche der Circular Economy vielfältige Anregungen bieten.

Nur wenn die Gestaltung resilienter Stadt‑/Wirtschaftsräume alle Möglichkeiten der Circular Economy integriert, lassen sich wahrhaft nachhaltige Zukunftsperspektiven schaffen.
